# The clinically extremely vulnerable to COVID: Identification and changes in healthcare while self-isolating (shielding) during the coronavirus pandemic.

**DOI:** 10.23889/ijpds.v7i3.1897

**Published:** 2022-08-25

**Authors:** Jessica Butler, Mintu Nath, Dimitra Blana, William Ball, Nicola Beech, Corri Black, Graham Osler, Sebastien Peytrignet, Magdalena Rzewuska, Katie Wilde, Artur Wozniak, Simon Sawhney

**Affiliations:** 1 University of Aberdeen; 2 NHS Grampian; 3 Health Foundation

## Objectives

In March 2020, Scottish government identified people clinically extremely vulnerable to COVID due to pre-existing health conditions. These people were advised to strictly self-isolate (shield) at home. We examined who was identified as clinically extremely vulnerable, how their healthcare changed during isolation, and whether this process exacerbated healthcare inequalities.

## Approach

We linked all individuals on the shielding register in NHS Grampian to their in-patient and out-patient healthcare records from 2015 through 2020. We analysed the method of patients’ identification as clinically extremely vulnerable (via an algorithmic NHS record scan or designated ad hoc by their care-providers). We measured out-patient, in-patient, and emergency healthcare attendances, and compared use rates between two 3-month periods before and during the first strict isolation period. We evaluated changes in care use between those shielding and the general non-shielding population, and differences between shielding sub-populations (by clinical reason for shielding, age, sex, and socio-economic deprivation).

## Results

The shielding register included 16,092 people (3% of the population). 42% of people on the register were not identified by national healthcare record screening, including the majority of cancer and immunocompromised patients. People added to the register by their care-providers were more likely to be young and less economically-deprived.

Shielders’ healthcare use decreased during isolation (rate compared to pre-isolation: 0.65 out-patient, 0.54 scheduled in-patient; 0.75 emergency in-patient; 0.71 A&E). However, people shielding had better maintained care than the non-shielding population (e.g. RR 2.9 for scheduled in-patient care). There were inequalities in whose scheduled care was maintained while shielding: younger people and those with cancer had significantly higher visit rates. However, there were no differences in care-preservation between men and women or between socioeconomic deprivation levels.

## Conclusion

The reliance on emergency care while shielding indicates that, overall, continuity of care for existing conditions was not optimal. However, there was notable success in maintaining care for cancer. We suggest that integrating demographic and primary care data would improve identification of the clinically vulnerable and help equitably prioritise care.

